# Drawing as a tool for investigating the nature of imagery representations of blind people: The case of the canonical size phenomenon

**DOI:** 10.3758/s13421-023-01491-7

**Published:** 2023-11-20

**Authors:** Magdalena Szubielska, Wojciech Kędziora, Paweł Augustynowicz, Delphine Picard

**Affiliations:** 1 Institute of Psychology, The John Paul II Catholic University of Lublin, Al. Racławickie 14, 20-950 Lublin, Poland; 2 Independent researcher, Lublin, Poland; 3 Aix Marseille University, PSYCLE, Aix en Provence, France

**Keywords:** Congenitally blind people, Adventitiously blind people, Drawings, Canonical size, Imagery

## Abstract

Several studies have shown that blind people, including those with congenital blindness, can use raised-line drawings, both for “reading” tactile graphics and for drawing unassisted. However, research on drawings produced by blind people has mainly been qualitative. The current experimental study was designed to investigate the under-researched issue of the size of drawings created by people with blindness. Participants (*N* = 59) varied in their visual status. Adventitiously blind people had previous visual experience and might use visual representations (e.g., when visualising objects in imagery/working memory). Congenitally blind people did not have any visual experience. The participant’s task was to draw from memory common objects that vary in size in the real world. The findings revealed that both groups of participants produced larger drawings of objects that have larger actual sizes. This means that the size of familiar objects is a property of blind people’s mental representations, regardless of their visual status. Our research also sheds light on the nature of the phenomenon of canonical size. Since we have found the canonical size effect in a group of people who are blind from birth, the assumption of the visual nature of this phenomenon – caused by the ocular-centric biases present in studies on drawing performance – should be revised.

## Introduction

### Blind people’s ability to use drawings

There is extensive literature on the ability to use pictorial representations and the creation of drawings by people with blindness. Psychological research in this area has addressed topics such as the recognition of geometrical forms (Heller et al., 2006) or the identification of everyday objects on tactile drawings produced using a variety of techniques (Heller, 1989; Heller et al., 1996; Lederman et al., 1990; Mascle et al., 2022; Pathak & Pring, 1989; Picard et al., 2013, 2014; Picard & Lebaz, 2012; Theurel et al., 2013; Vinter et al., 2020) by blind participants of a wide variety of ages. In addition, research has dealt with relationships between haptic exploratory strategies and the recognition of two-dimensional embossed pictures or drawing performance (D’Angiulli et al., 1998; Magee & Kennedy, 1980; Vinter et al., 2012).

When it comes to the production of raised-line drawings by participants with blindness, the analyses have mainly considered the recognisability and quality of the drawings – assessed by researchers on their own or by judges (D’Angiulli & Maggi, 2003; Kennedy, 1993; Millar, 1975; Szubielska et al., 2016; Szubielska, Niestorowicz, & Marek, 2019b; Wu et al., 2022; see also Szubielska, Imbir, et al., 2020), including in particular the occurrence of “visual conventions” (e.g., perspective shortcuts, occlusion) in the drawings of people deprived of visual experience (Carboni et al., 2021; Kennedy, 2003; Kennedy & Juricevic, 2003, 2006a, 2006b, 2008). Importantly, the recognisability and formal features (e.g., the use of contour lines) of the drawings produced under haptic control seem to depend on practice at drawing and the severity of sight impairment. Overall, the quality of drawings might increase with the drawing experience of participants with severe visual impairment, and be positively related to their ability to use mental visual imagery (D’Angiulli & Maggi, 2003; I & Shiu, 2010; Vinter et al., 2018; Wu et al., 2020). In other words, drawing appears to be more challenging for people who are blind from birth than for late blind individuals. Nevertheless, as Kennedy (e.g., 1993) argues, the greater difficulty in producing drawings encountered by people with congenital blindness may be due to a lack of practice in drawing rather than the lack of vision per se. Such a point of view, built on a study conducted among children with visual impairment, is shared by Vinter et al. (2018). In addition, some studies have focused on a qualitative analysis of the metaphoric aspects of drawings, such as depicting movement, sounds or mental events (Kennedy, 2008, 2009, 2013, 2014a, 2014b; Kennedy & Merkas, 2000; see also D’Angiulli & Maggi, 2003).

On the other hand, research on the quantitative characteristics of drawings produced by blind people is scarce. To our knowledge, only one study has so far tested the quantitative feature of drawing size (Wu et al., 2022) – but only in the context of recognising tactile drawings, not their creation by participants who are blind. In this study, congenitally blind participants needed more time to identify large- and medium-scale graphics than small-scale ones, probably due to similarities between the size of small-scale pictures and the actual objects (hence, the size was familiar, and the objects were easier to identify). It is possible that this finding was related to the experience of using tactile graphics by blind participants – the standards for creating these types of graphics recommend designing hand-sized embossed pictures (e.g., Edman, 1992). Visual experience and familiarity with using haptic exploration for recognising images (sighted people lack such experience) possibly modify the optimal size for recognising tactile drawings by touch, as the opposite results were obtained among blindfolded sighted people – in this case, the larger embossed graphics were more recognisable than the smaller ones (Kennedy & Bai, 2002; Wijntjes et al., 2008). In another study involving people blind since birth, it was found that the recognisability of the drawings produced from memory under haptic control depended on the actual size of the physical objects – more recognisable drawings were created for larger objects (furniture size) than for smaller objects (hand size) (Szubielska, Niestorowicz, & Marek, 2019b). Unfortunately, this study did not explore the size of the drawings produced by the participants. As we will discuss later, looking at drawing size in cases of blindness is interesting for several reasons, including testing the presence of a canonical size effect, which was first discovered in the visual mode (Konkle & Oliva, 2011).

Summing up, to date the research on the use of drawings by blind people has placed much more emphasis on analysing qualitative rather than quantitative features. More specifically, when it comes to the active production of tactile pictures by blind people, studies focused on drawing quality issues – mainly their recognisability, resulting from the (im)perfection of shape.

### The canonical size phenomenon – Evidence from the visual and haptic domains

One of the more interesting properties of drawings by people with blindness, to date overlooked in the literature, is the size of the drawings created. This feature was first analysed in the drawings of sighted people made under visual control (Konkle & Oliva, 2011), which showed that the size of the drawing depends on the actual size of the object being drawn. More precisely, the larger the actual object is, the larger the area of a sheet of paper is occupied when drawing this single object. This effect was referred to as the *visual canonical size phenomenon*. The neural correlates of differentiating the objects’ real-world size were further found in the ventral temporal cortex (Konkle & Oliva, 2012b).

Research investigating the phenomenon of canonical visual size has not only used the task of drawing from memory but also a mental imagery paradigm (the size at which objects were imagined within the computer monitor’s frame) and a perception paradigm (the participant’s task was to view images of real-world objects and determine the size at which they looked best) (Konkle & Oliva, 2011). The results obtained in all the research paradigms analysed show a preference for representing objects in the frame as having a larger size, the larger the objects are in reality. This finding suggests that size information is a property of an object’s mental representation. However, so far, the canonical size phenomenon has only been tested in adults with normal or corrected-to-normal vision. Interestingly, recent research by Chen et al. (2022) showed similar visual size preferences concerning hardly recognisable objects (i.e., pictures of so-called texforms, which maintain local texture and rough contour information). Participants consistently selected the texform presented at the canonical visual size as more aesthetically appealing. Furthermore, using a modified Stroop (1935) task, Konkle and Oliva (2012a) provided evidence that the objects’ familiar size is accessed automatically by sighted people when viewing images of objects.

Although the canonical size effect was initially assumed as visual (Konkle & Oliva, 2011), recent studies conducted in the visual and haptic domains (Szubielska et al., 2022; Szubielska & Wojtasiński, 2021; Szubielska, Wojtasiński et al., 2020) have questioned the visual character of this phenomenon. In all these recent studies, the canonical size effect was investigated using the task of drawing from memory among participants without visual impairments. Although these studies found that larger drawings were produced in the visual than in the blindfolded condition (Szubielska et al., 2022; Szubielska, Wojtasiński et al., 2020), they revealed the canonical size effect in both the visual and the haptic domains. Intriguingly, the canonical size effect was revealed even when blindfolded participants drew on ordinary paper sheets, which drastically reduced the possibility of haptic control of the drawing that was created (which in turn was possible in the case of drawing on special foils for raised-line drawings, where participants controlled the in-progress drawing with their non-dominant hand) (Szubielska et al., 2022). However, these aforementioned studies on the canonical size effect in the haptic domain were again conducted among normally sighted participants, thus revealing the phenomenon of canonical size under blindfolded conditions is insufficient evidence for this phenomenon’s non-visual (abstract or multimodal) nature. After all, it is typical for sighted individuals to visualise spatial stimuli (Pantelides et al., 2016; Szubielska, 2014; Vanlierde & Wanet-Defalque, 2004), so the participants could have used visual mental images of objects placed in imagined frames when performing the blindfold drawing task.

The potential visual nature of the phenomenon of interest in this paper might be confirmed by testing people with blindness who (as we argued beforehand) have the ability to draw, but their mental representations are non-visual. If the phenomenon of canonical size is uniquely visual, it should not be manifest in congenitally blind individuals. However, previous studies suggest that the phenomenon may be spatial rather than visual (Szubielska et al., 2022; Szubielska & Wojtasiński, 2021; Szubielska, Wojtasiński, et al., 2020). Moreover, size is, by definition, a spatial property, and spatial cognition, being modality-independent, may occur via domains other than sight (e.g., touching objects and even verbal descriptions – for a discussion, see Loomis et al., 2013). Therefore, spatial information and spatial mental representation are not unique to sighted people or reliant on visual imagery (for a literature review see, e.g., Cattaneo et al., 2008; Ricciardi et al., 2014). Consequently, the canonical size phenomenon might be manifested in people without visual experience.

### Mental imagery abilities of congenitally blind and adventitiously blind people

Researchers who investigated blind participants’ spatial abilities or mental imagery suggest that human spatial representations and underpinned cortical organisation might be visually independent. Likova (2012) argues that the primary visual cortex may provide for a modality-independent (possibly amodal) sketchpad function of the working memory, a function that is needed to process mental images. Others (e.g., Cattaneo et al., 2008; Ricciardi et al., 2014), based on the literature on the structural and the functional exploration of the brain of people with normal vision and those blind from birth, opt for a supramodal cortical functional architecture (since similar cortical networks seem to subtend visual and non-visual cognition of spatial properties both in sighted and congenitally blind individuals). Supramodality means that spatial information is processed by distinct cortical areas/networks independently from the sensory modality that carries information to the brain. Furthermore, the findings from behavioural studies using classic mental imagery paradigms (in which visual imagery used to be considered to be critically involved) compared spatial cognition in sighted and congenitally blind participants and showed that the classic mental imagery effects (e.g., scanning effect: Blanco & Travieso, 2003; Iachini & Ruggiero, 2010; or rotation effect: Marmor & Zaback, 1976) are also revealed in people who lack visual experience (for a review, see Cattaneo et al., 2008). To sum up, spatial mental representation in general and drawing processing in particular seem equally possible in people who are sighted and congenitally totally blind because mental imagery does not need to be visual (it may have a more abstract, spatial character).

Psychologists have long emphasised the relationships between cognition, knowledge and drawing (e.g., Freeman & Cox, 1985; Jolley, 2010; Kennedy, 1993; Luquet, 2001; Piaget, 1926, 1929; van Sommers, 1984; see also Konkle & Oliva, 2011). In the case of congenitally blind people, drawing from memory might be treated as an indicator of an ability to produce mental imagery (Szubielska et al., 2016) or the operation of the modality-independent spatial sketchpad of working memory (as suggested by Likova, 2012).

Drawing by congenitally blind people contributes to involving brain areas commonly associated with vision and visual imagery representations (Amedi et al., 2008; Cacciamani & Likova, 2017; 2021). However, of course, the results of these functional brain activity studies do not provide evidence that the mental representations of congenitally blind people are visual^1^ (for discussion, see Likova, 2012) since – as mentioned before – spatial cognition seems to be modality-independent and drawing abilities refer to spatial cognition rather than visual perception.

Thus, congenitally blind and adventitiously blind people are similar in that they can use spatial imagery. At the same time, only adventitiously blind individuals can visualize spatial stimuli (Vanlierde & Wanet-Defalque, 2004; see also Picard et al., 2010). Some suggest that the ability to visualize is related to a preference for a particular frame of reference when representing spatial information (e.g., Toroj & Szubielska, 2011). In this vein, some studies have shown that people who are blind from birth predominantly use egocentric (body-centred) reference frames and adventitiously blind people – allocentric ones (Pasqualotto et al., 2013; Pasqualotto & Proulx, 2012; Ruggiero et al., 2012, 2022; Toroj & Szubielska, 2011). Therefore, when constructing mental representations, a congenitally blind individual refers an object to the body, and an adventitiously blind refers an object to another object (e.g., the object to the imagined boundaries in which it is placed). However, there is also evidence that adults (Chiesa et al., 2017; Schmidt et al., 2013) and children (Martolini et al., 2021) who are congenitally totally blind use allocentric spatial information where needed and (similar to sighted participants) spontaneously evoke allocentric spatial frames to perform spatial tasks; for instance, they adopt an allocentric survey strategy when mentally representing a town environment (for a review, see Ottink et al., 2022).

To date, it has not been established whether the size of actual objects is a property of blind people’s mental representations. Nevertheless, there are reports that congenitally blind participants find it challenging to accurately represent the angular size of an object at different distances from the observer (Arditi et al., 1988; Vanlierde & Wanet-Defalque, 2005; for a contrary finding, see Wnuczko & Kennedy, 2014). In turn, late blind people seem not to experience similar difficulties (Vanlierde & Wanet-Defalque, 2005). Perhaps late blind people not only estimate the angular size of objects imagined at varied distances more accurately than congenitally blind people, but they also more accurately represent the size of familiar everyday objects. However, some researchers argue that the imagery abilities, including those required to perform complex spatial tasks, of people who are blind from birth are underestimated (Eardley & Pring, 2007). Due to the higher mental imagery abilities of congenitally blind people than is stereotypically believed, among other things, it is possible to effectively teach mathematics (including geometry) to blind students (Ostad, 1989), and spontaneous drawing development in congenitally blind children is possible (D’Angiulli & Maggi, 2003).

#### The current study

The present study is designed to investigate whether the size of real-world objects is a property of the mental representations of adults with blindness, especially those blind from birth. In other words, we explored the canonical size effect (Konkle & Oliva, 2011) among adventitiously and congenitally blind adults, using the task of drawing familiar objects from memory. Like in the most recent study conducted in the haptic domain in this area (Szubielska et al., 2022), we used two materials for drawing – plain paper and special foils for producing raised-line drawings. The topics of the drawings were real world objects of eight different sizes.

## Method

### Participants

Fifty-nine adult participants with blindness participated in the study (28 totally blind, 31 with a sense of light) (initially, 64 blind participants were tested, but data from five individuals were rejected due to their uncertain visual status regarding visual memories – these participants lost their sight in early childhood). Two groups of people with blindness were tested: (a) congenitally blind (CB) (*n* = 30; 18 males, 12 females; 28 right-handed; aged 21–62 years, *M* = 34.80, *SD* = 12.34), i.e., those who have not seen since the beginning of their lives and (b) adventitiously blind (AB) (*n* = 29; 17 males, 12 females; 27 right-handed; aged 18–61 years, *M* = 40.83 years, *SD* = 12.36), i.e., those who lost their sight during their lives (when aged between 4 and 59 years; *M* = 20.10, *SD* = 13.72) and had visual memories. None of the participants had visual form perception. More than half of the participants in each group had a university degree (CB: 53%, AB: 52%), and the rest had, at most, a secondary education. None of the participants had a combination of disabilities. Detailed information about the participants is presented in Appendix 1 Table 4.

### Sample size

The sample size was based on previous studies on the canonical size effect in the haptic domain (Szubielska et al., 2022; Szubielska, Wojtasiński et al., 2020). A priori power analyses using G-Power 3.1. (Faul et al., 2007) yielded the conclusion that, based on a significance level of *p* < .05 and a power of .95 (here and throughout) and the effect size of *f* = 1.08 (Szubielska et al., 2022), *N* = 8 participants would be needed to detect a within-participants effect of size rank in a repeated measures analysis of variance (ANOVA). In addition, the necessary sample size to detect a between-participants effect of visual status was estimated to be *N* = 28 – based on the effect size of *f* = 0.65, and to detect between-within interaction between size rank and visual status was estimated to be *N* = 12 – based on the effect size of *f* = 0.48 (Szubielska, Wojtasiński et al., 2020). Due to possible variations in experience of drawing within the group of people with blindness, we decided to test more than 28 participants.

### Materials

Like in the previous studies on the canonical size effect in the haptic domain (Szubielska et al., 2022; Szubielska & Wojtasiński, 2021; Szubielska, Wojtasiński et al., 2020), we used a Swedish raised-line drawing kit (i.e., a rubber mat with an A4 foil for producing embossed drawings), sheets of standard A4 paper, and sharpened pencils.

### Design

We used a mixed design, with size rank (8) and material used (2) as within-participant variables and participants’ visual status (2) and order of drawing (2) as between-participant variables.

### Procedure

Participants were tested individually in two blocks – using foil or paper for producing drawings. The order of these blocks (drawing on foil first vs. on paper first) was counterbalanced across participants. If needed, a short break was taken between blocks.

In the beginning, participants were familiarised with the Swedish raised-line drawing kit. Then, participants were asked to use their non-dominant hand to explore the embossed shapes produced during the drawing process (to have haptic feedback in the foil condition). Then, they were informed about the task, i.e., they were asked to draw from memory without a time limit a single object per sheet of paper/foil orientated horizontally (no turning of the sheet while drawing). Both paper and foil sheets had an A4 format. At no point in the experiment was it suggested what size the drawing should be, nor was the actual size of the objects to be drawn mentioned. Furthermore, none of the participants asked about the drawing size that we would expect in the study.

In each block, participants drew from memory, in random order: (1) key, (2) apple, (3) shoe, (4) backpack, (5) dog, (6) floor lamp, (7) car, (8) house (the same topics as were used by Szubielska et al., 2022, for drawings). These subsequent topics (and their numbers) correspond to objects that can be ranked due to their increasing size in the real world (see Konkle & Oliva, 2011). After producing the drawing, participants were asked about any additional objects that potentially were added to the object which was the subject of the drawing (unless the participants spontaneously provided such information while producing the picture).

After the drawing from memory task, participants were asked to provide their demographic characteristics (gender, age, level of education), their visual impairment history and severity and experience in producing drawings (“How often have you drawn?”) and familiarity with embossed graphics (“How often have you used embossed graphics?”; possible responses for both questions: “never”, “rarely”, “sometimes”, “often”, “very often”).

The study lasted 25 min on average per participant.

### Data coding

The indicator of the drawn size of the object (in millimetres) was measured by the length of the diagonal of the rectangle bounding the drawing (like in previous studies in this field: Konkle & Oliva, 2011; Szubielska et al., 2022; Szubielska & Wojtasiński, 2021; Szubielska, Wojtasiński et al., 2020). In line with the previous studies, extraneous objects were ignored (e.g., a fence next to the house; to identify these extraneous objects, we asked the participants about the presence of additional objects after they had made the drawings – “Have you drawn anything else in addition?”). Only the relevant object of interest was bounded around by a rectangle. As in the study by Szubielska et al. (2022), all drawings were scanned at a fixed resolution, the rectangle boundaries were determined using the Photoshop program, and custom software converted the dimension of the rectangle into millimetres and then – into diagonals.

## Results

### Preliminary analyses

Using Pearson’s chi-square test, we compared whether participants who were blind from birth and adventitiously blind differed in their drawing experience and familiarity with convex graphics. Both calculations did not yield significant differences between congenitally and adventitiously blind – respectively, χ^2^(4) = 3.75, *p* = .441, χ^2^(4) = 7.28, *p* = .122. Overall, 46% of participants declared that they had never drawn (for detailed information on the drawing experience and familiarity with convex graphics of both groups of blind participants, see Table 1).

**Table 1:** Characteristics of the congenitally blind (CB) and adventitiously blind (AB) groups toward drawing experience and familiarity with convex graphics

	“How often have you drawn?”		“How often have you used embossed graphics?”	
	CB	AB	CB	AB
Never	50.0% (*n* = 15)	41.4% (*n* = 12)	0.0% (*n* = 0)	17.2% (*n* = 5)
Rarely	40.0% (*n* = 12)	31.0% (*n* = 9)	20.0% (*n* = 6)	27.6% (*n* = 8)
Sometimes	3.3% (*n* = 1)	17.2% (*n* = 5)	23.3% (*n* = 7)	13.8% (*n* = 4)
Often	3.3% (*n* = 1)	3.4% (*n* = 1)	43.3% (*n* = 13)	27.6% (*n* = 8)
Very often	3.3% (*n* = 1)	6.9% (*n* = 2)	13.3% (*n* = 4)	13.8% (*n* = 4)

### Investigating the canonical size effect

**Fig. 1:**
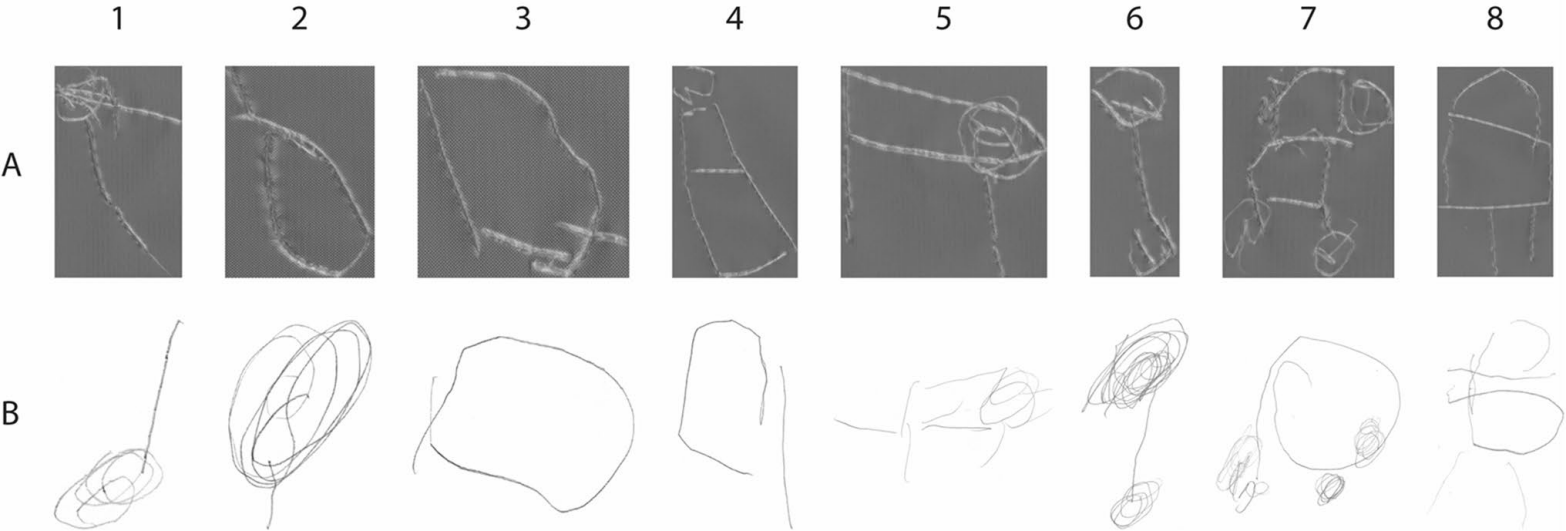
Examples of drawings produced on foil (**A**) and paper (**B**) by the same congenitally blind participant. Drawings show key (1), apple (2), shoe (3), backpack (4), dog (5), floor lamp (6), car (7), and house (8). All drawings are presented trimmed to the drawing boundaries

Table 2 presents descriptive statistics of drawn size for all experimental conditions. Examples of drawings made on foil and paper are shown in Fig. 1.

**Table 2:** Mean drawn size as a function of size rank, presented for each material and participants’ visual status (congenitally blind and adventitiously blind), separately for each drawing order. Standard deviations are presented in parentheses

Size rank	Drawing order	Material			
		Foil		Paper	
		Visual status			
		CB	AB	CB	AB
1	Foil first	70.20 (32.53)	104.04 (52.45)	61.15 (32.68)	79.86 (35.26)
	Paper first	94.72 (45.07)	93.46 (57.63)	68.24 (45.72)	82.97 (63.23)
2	Foil first	94.52 (84.10)	115.71 (48.73)	60.57 (27.38)	124.91 (83.17)
	Paper first	93.94 (57.66)	111.46 (70.87)	86.35 (60.36)	94.63 (53.95)
3	Foil first	93.21 (28.29)	96.28 (65.96)	77.52 (33.00)	91.44 (62.22)
	Paper first	108.08 (69.15)	119.75 (79.56)	98.78 (57.93)	106.56 (67.82)
4	Foil first	132.80 (58.19)	121.54 (81.40)	101.00 (57.72)	106.99 (49.73)
	Paper first	152.62 (71.84)	107.16 (77.09)	136.43 (63.75)	96.91 (58.79)
5	Foil first	129.68 (62.19)	134.31 (52.82)	118.13 (69.75)	126.16 (42.59)
	Paper first	171.62 (59.83)	147.91 (88.99)	135.48 (52.78)	142.28 (85.04)
6	Foil first	110.79 (45.46)	142.36 (61.13)	103.79 (43.64)	125.98 (57.82)
	Paper first	149.93 (58.39)	131.26 (66.00)	119.17 (56.37)	122.95 (52.59)
7	Foil first	133.39 (52.20)	186.73 (70.32)	128.84 (66.22)	144.94 (63.75)
	Paper first	179.55 (72.36)	174.74 (87.12)	133.56 (64.80)	148.04 (79.96)
8	Foil first	156.09 (78.73)	164.67 (66.73)	140.34 (80.31)	164.70 (68.38)
	Paper first	198.25 (85.52)	181.22 (87.43)	171.04 (80.91)	155.16 (75.83)

The drawn sizes were measured as diagonals of the drawing boundaries (in mm). Size rank values refer to the following objects: 1 – key, 2 – apple, 3 – shoe, 4 – backpack, 5 – dog, 6 – floor lamp, 7 – car, 8 – house

To investigate whether participants drew objects that are larger in the real world as larger and whether this depended on their visual experience, we computed an analysis of variance with the within-participant variables of size rank (1 – key vs. 2 – apple vs. 3 – shoe vs. 4 – backpack vs. 5 – dog vs. 6 – floor lamp vs. 7 – car vs. 8 – house) and material used (foil vs. paper), and the between-participant variables of participants’ visual status (congenitally blind vs. adventitiously blind) and order of drawing (on foil first vs. on paper first). We used the Greenhouse-Geiser-corrected values in case of violations of the sphericity assumption.

**Fig. 2:**
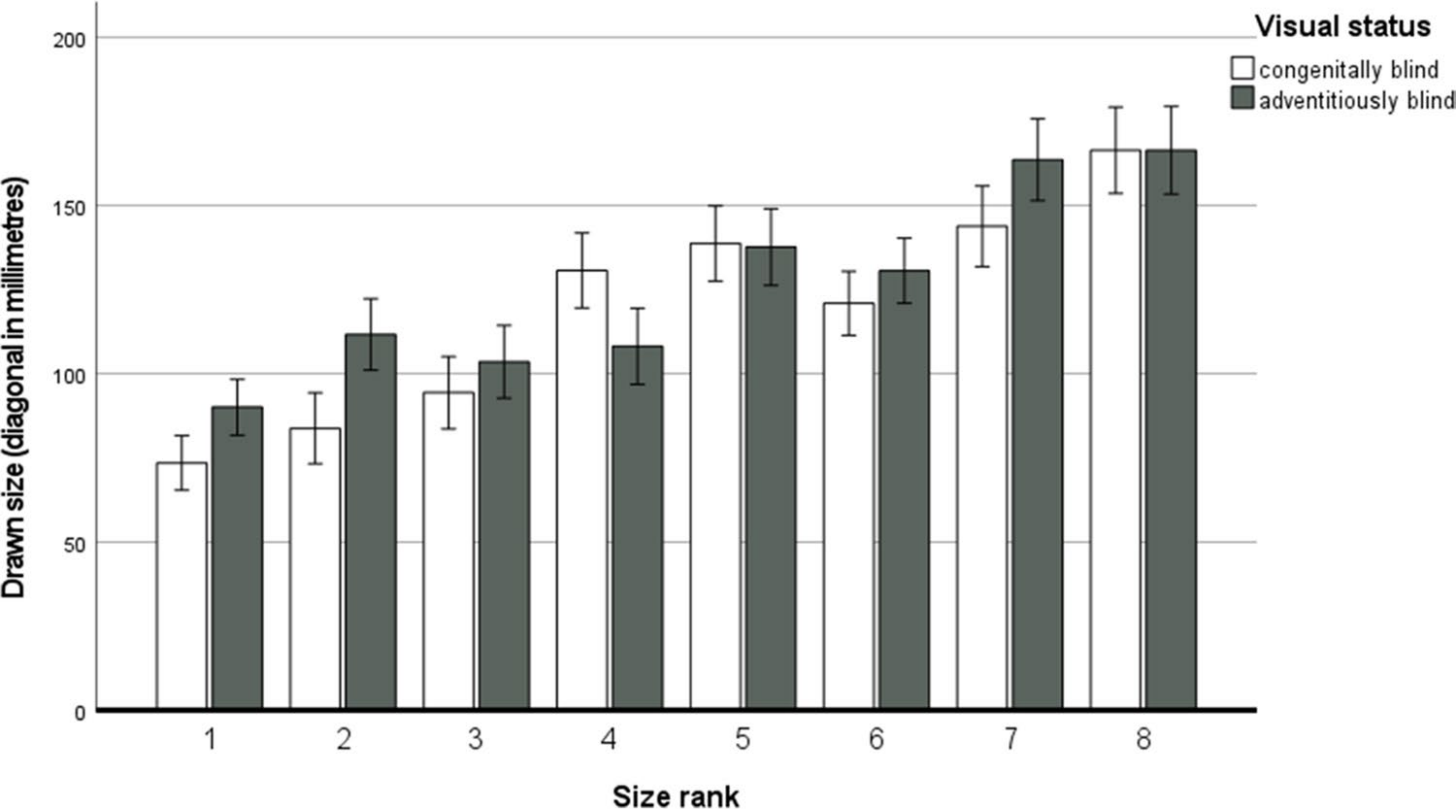
Drawn size as a function of visual status and size rank. Error bars indicate ±1 standard error. Size rank values refer to the following objects: 1 – key, 2 – apple, 3 – shoe, 4 – backpack, 5 – dog, 6 – floor lamp, 7 – car, 8 – house

This ANOVA showed a significant main effect of size rank – best explained by the linear function, *F*(1, 55) = 159.75, *p* < .001, *η*_*p*_^*2*^ = .74, a significant main effect of material (participants produced larger drawings on foil, *M* = 131.31, *SE* = 7.10, than paper, *M* = 114.21, *SE* = 6.50), and a significant interaction between size rank and visual status (see Fig. 2; for all inferential statistics, see Table 3). These effects were qualified by a significant four-way interaction between size rank, material, visual status, and drawing order (see Table 3). To dissect this interaction, we conducted follow-up ANOVAs for each visual status (congenitally blind and adventitiously blind) separately, with the within-participant variables of size rank and material used and the between-participant variable of drawing order.

**Table 3:** Results of the ANOVA (main effects and interactions) for drawn size as a dependent variable

	ANOVA effects
Size rank	***F*(4.98, 273.98) = 44.80,** ***p*** **< .001,** ***η***_***p***_^***2***^ **= .45**
Material	***F*(1, 55) = 20.99,** ***p*** **< .001,** ***η***_***p***_^***2***^ **= .28**
Visual status	*F*(1, 55) = 0.32, *p* = .573, *η*_*p*_^*2*^ = .01
Drawing order	*F*(1, 55) = 0.79, *p* = .379, *η*_*p*_^*2*^ = .01
Size rank **×** Material	*F*(5.02, 275.88) = 1.14, *p* = .336, *η*_*p*_^*2*^ = .02
Size rank × Visual status	***F*(4.98, 273.98) = 3.15,** ***p*** **= .009,** ***η***_***p***_^***2***^ **= .05**
Size rank × Drawing order	*F*(4.98, 273.98) = 0.89, *p* = .486, *η*_*p*_^*2*^ = .02
Material × Visual status	*F*(1, 55) = 0.86, *p* = .357, *η*_*p*_^*2*^ = .02
Material × Drawing order	*F*(1, 55) = 0.53, *p* = .469, *η*_*p*_^*2*^ = .01
Visual status × Drawing order	*F*(1, 55) = 0.91, *p* = .344, *η*_*p*_^*2*^ = .02
Size rank **×** Material × Visual status	*F*(5.02, 275.88) = 0.66, *p* = .653, *η*_*p*_^*2*^ = .01
Size rank **×** Material × Drawing order	*F*(5.02, 275.88) = 0.72, *p* = .612, *η*_*p*_^*2*^ = .01
Size rank **×** Visual status × Drawing order	*F*(4.98, 273.98) = 0.58, *p* = .715, *η*_*p*_^*2*^ = .01
Material × Visual status × Drawing order	*F*(1, 55) = 0.20, *p* = .656, *η*_*p*_^*2*^ = .00
Size rank **×** Material × Visual status × Drawing order	***F*(5.02, 275.88) = 2.75,** ***p*** **= .019,** ***η***_***p***_^***2***^ **= .05**

Significant effects are indicated in bold. The samples included in this analysis vary by visual status and drawing order (CB: *n* = 14 in the “foil first” condition, *n* = 16 in the “paper first” condition; AB: *n* = 15 in the “foil first” condition, *n* = 14 in the “paper first” condition)

Among congenitally blind participants, the ANOVA yielded a main effect of material, *F*(1, 28) = 16.73, *p* < .001, *η*_*p*_^*2*^ = .37 – because participants produced larger drawings on foil (*M* = 129.34, *SE* = 9.23) than paper (*M* = 108.77, *SE* = 8.73). The main effect of size rank was also significant, *F*(4.40, 123.13) = 32.08, *p* < .001, *η*_*p*_^*2*^ = .53. This effect was best explained by the linear function, *F*(1, 28) = 99.44, *p* < .001, *η*_*p*_^*2*^ = .78. The size of the drawing increased as the size rank value increased (see Fig. 2). The remaining main effects and interactions did not reach significance (all ps > .065).

The pattern of results was similar for the adventitiously blind group. The main effect of material was significant, *F*(1, 27) = 6.08, *p* = .020, *η*_*p*_^*2*^ = .18 – due to larger drawings being produced on foil (*M* = 133.29, *SE* = 10.82) than on paper (*M* = 119.65, *SE* = 9.66). In addition, the main effect of size rank was also significant, *F*(4.87, 131.49) = 17.83, *p* < .001, *η*_*p*_^*2*^ = .40 and best explained by a linear function *F*(1, 27) = 63.73, *p* < .001, η_*p*_^*2*^ = .70. Adventitiously blind participants produced larger drawings with increasing size rank value (see Figure 2). The other effects were non-significant (all ps > .189).

Since one may argue that the paper condition is problematic from the ecological validity point of view, we calculated additional analysis only on foil data (see Appendix 2). These findings of the ANOVA are similar to those obtained for all data, i.e., we yielded a statistically significant size rank effect best described by the linear function for congenitally blind and adventitiously blind participants.

## Discussion

In the current study, we investigated the canonical size phenomenon (Konkle & Oliva, 2011) in adventitiously and congenitally blind participants. Their task was to draw common objects (of eight different sizes in the real world) from memory in two conditions – on paper or foil for raised-line drawings.

In both groups of blind participants, we found a similar pattern of results, i.e., increasing drawn size for objects that have larger real-world sizes. Importantly, the main effect of size rank was best explained by a linear function. Previous studies have found the same pattern of results among sighted adult participants who performed the drawing task in the visual or haptic domains (Konkle & Oliva, 2011; Szubielska et al., 2022; Szubielska & Wojtasiński, 2021; Szubielska, Wojtasiński et al., 2020). That means that the size of the real-world objects is a property of the mental representations of adults with blindness, regardless of their visual status. This result may also suggest that object size is a defining property of mental representations of familiar objects and that knowledge of object size may be gained from different learning procedures, like direct sensory experience (not only visual but probably also haptic) or more abstract knowledge.

Moreover, this result suggests that blind adults, including those who are blind from birth, used rather allocentric reference frames when performing the task, which relate the drawn size of the particular object to the size of the frame determined by the surface of the sheet of paper/foil rather than body-centred reference frames. If the participants had used egocentric strategies based on their previous experience in tactile graphics, all drawings of objects would have been similar, approximately hand size. All of the congenitally blind participants had (at least rare) previous experience of using tactile graphics, and guidelines for depicting objects on tactile graphics recommend hand size (Edman, 1992). If, on the other hand, the participants had referred to their experience of touching actual objects, the shoe should fill almost the entire sheet of paper, while objects like a backpack and larger would go beyond the A4 size sheet. Nevertheless, the congenitally blind participants using sheets of paper/foil of this size produced drawings that generally were not larger than the hand but varied in size according to the objects’ sizes in the real world. Hence, our findings contrast reports from previous studies, which suggested that congenitally blind people prefer using egocentric reference frames (Pasqualotto et al., 2013; Pasqualotto & Proulx, 2012; Ruggiero et al., 2012, 2022; Toroj & Szubielska, 2011) and are in line with those which showed use of an allocentric reference frame in congenitally blind individuals (Chiesa et al., 2017; Martolini et al., 2021; Ottink et al., 2022; Schmidt et al., 2013).

The results obtained in the present study also provide evidence that people who are blind from birth can correctly estimate angular size when drawing (other paradigms of testing angular size representation revealed opposite results, Arditi et al., 1988; Vanlierde & Wanet-Defalque, 2005) and scale sizes (for similar findings in the task of the spatial scaling of maps, see Szubielska, Möhring, & Szewczyk, 2019a). Hence, our research also shows that people who are blind from birth do not ignore the size of the objects they imagine, as has been suggested in studies on mental majorization of abstract shapes (the process of majorization is defined as mental transformation requiring enlargement of the object of imagery representation) (Szubielska, 2015). However, perhaps there are differences between blind participants in representing the size in the case of real and abstract objects. Intriguingly, we found the canonical size phenomenon in participants with congenital blindness even though only half of them declared some drawing experience and that their drawings were hardly recognisable (see Fig. 1). This may mean that angular size is represented more accurately in the congenitally blind person’s mind than the two-dimensional shape of three-dimensional objects.

The results also revealed that both congenitally and adventitiously blind participants produced larger drawings on foil than paper. Previous research, in which the type of material (film vs. paper) was manipulated, did not reveal a similar finding (Szubielska et al., 2022). This may mean that the ability to have perceptual control during the drawing process is vital for the size of the drawing. Smaller drawings are created when perceptual feedback (haptic or visual – in Szubielska et al.’s (2022) study, sighted participants produced larger drawings in the visual than blindfolded condition) is limited.

In addition to revealing that blind people represent the size of familiar objects in imagery, our research also brings novel findings on the nature of the phenomenon of canonical size. This phenomenon was initially claimed to be visual due to being tested in the visual domain and linked to visual perception (Konkle & Oliva, 2011, see also Chen et al., 2022; Konkle & Oliva, 2012b). However, in this study, we found a canonical size effect in participants with congenital blindness who cannot use visual representations at all (Blanco & Travieso, 2003; Likova, 2012; Picard et al., 2010; Szubielska, 2014; Vanlierde & Wanet-Defalque, 2004). Therefore, our study negates the assumption of the phenomenon’s purely visual nature.

Consequently, it can be argued that the canonical size phenomenon itself is spatial, not visual. This conclusion is in line with the concept of a supramodal spatial system and an amodal spatial function (e.g., Cattaneo et al., 2008; Likova, 2012; Ricciardi et al., 2014; Wolbers et al., 2011). Furthermore, our findings support the concept of functional equivalence of spatial representations from touch and vision (Giudice et al., 2011; Ottink et al., 2021) in the sense that touch, in a similar way to vision, allows the acquisition and use of implicit knowledge of the sizes of everyday objects.

## Limitations

We consider the main limitation of our study to be that the canonical size effect was tested using only one task – drawing from memory. To further confirm and generalise the amodal character of the canonical size phenomenon, it would be helpful to investigate it among congenitally blind participants performing other tasks – the imagery and perceptual tasks used by Konkle and Oliva (2011) – but adapted to the haptic domain.

Another limitation is that a floor lamp (which refers to size rank 6) might have been more challenging to draw by blind participants than the other objects considered in this study. Although lamps seem useless for blind people in everyday life, at the same time, none of the participants mentioned to us that they did not know what such a lamp is or looked like. Moreover, the participants were not instructed to draw a lamp when switched on, and the spatial properties of a lamp (shape, size) can be learned as much through sight as through touch. However, for better control of familiarity, this variable might have been measured by asking the participants to rate familiarity with an object drawn in the study after the drawing phase – concerning the floor lamp and all other objects included in the study.

One may also consider the lack of the ecological validity of the paper condition as a limitation. On the one hand, perceptual control in this condition is minimal (since haptic feedback is unavailable, but proprioceptive information still is available – for a discussion, see Szubielska et al., 2022). On the other hand, similar procedures (i.e., drawing without haptic feedback) were adopted in other studies on drawing among blind participants (e.g., Likova, 2012), and one of the participants in our study spontaneously declared that she often drew on paper for her child. In addition, and most importantly, the analysis performed excluding the data collected in the paper condition yielded similar results to the analysis performed on all the data. Notably, the canonical size effect was confirmed in both analyses.

## Conclusions

Our quantitative study on the drawn size of familiar objects drawn from memory has shown that size is a feature of mental representations of real-world objects among blind people, including those with congenital blindness. More precisely, our findings suggest that late and congenitally blind people mentally represent objects as larger when they have larger actual physical sizes. From a theoretical perspective, our study contributes to correcting the ocular-centric bias underpinning conclusions about the visual nature of the canonical size phenomenon. The findings obtained among congenitally blind participants allow us to assume that the nature of this phenomenon is spatial, not visual.

## Appendix 1

**Table 4:** Details of the congenitally blind and adventitiously blind participants

Participant	Age*	Gender*	Blindness aetiology*	Onset of blindness (years living without sight)*	Diffuse light perception*
*Congenitally blind (CB)*					
CB1	36	M	Retinoblastoma	Birth	No
CB2	48	M	Birth injury	Birth	No
CB3	25	F	Retinopathy of prematurity	Birth	Yes
CB4	29	M	Unknown	Birth	Yes
CB5	29	M	Retinoblastoma	Birth	No
CB6	62	M	Congenital defect	Birth	No
CB7	30	M	Retinopathy of prematurity	Birth	Yes
CB8	25	F	Retinopathy of prematurity	Birth	No
CB9	26	M	Retinopathy of prematurity	Birth	No
CB10	42	M	Toxoplasmosis	Birth	Yes
CB11	21	F	Eyeball underdevelopment	Birth	Yes
CB12	24	F	Optic nerve hypoplasia	Birth	Yes
CB13	21	F	Optic nerve hypoplasia	Birth	Yes
CB14	21	F	Retinal degeneration	Birth	No
CB15	56	F	Retinal degeneration	Birth	Yes
CB16	60	M	Optic nerve hypoplasia	Birth	No
CB17	27	M	Retinopathy of prematurity	Birth	Yes
CB18	35	M	Genetic disease	Birth	No
CB19	31	F	Optic nerve atrophy	Birth	No
CB20	60	F	Retinopathy of prematurity	Birth	No
CB21	36	M	Retinopathy of prematurity	Birth	No
CB22	27	M	Congenital defect	Birth	Yes
CB23	22	F	Congenital defect	Birth	Yes
CB24	39	M	Retinopathy of prematurity	Birth	Yes
CB25	30	M	Retinopathy of prematurity	Birth	Yes
CB26	43	M	Retinopathy of prematurity	Birth	No
CB27	36	F	Unknown	Birth	No
CB28	24	M	Retinopathy of prematurity	Birth	Yes
CB29	32	F	Retinopathy of prematurity	Birth	Yes
CB30	47	M	Congenital defect	Birth	Yes
*Adventitiously blind (AB)*					
AB1	48	M	Disease	6	No
AB2	28	M	Toxoplasmosis	12	No
AB3	61	M	Injury	10	No
AB4	61	M	Optic nerve atrophy	15	No
AB5	36	M	Injury	13	No
AB6	50	F	Disease	39	No
AB7	36	F	Injury	11	Yes
AB8	47	M	Injury	36	No
AB9	52	M	Retinal degeneration	35	No
AB10	41	F	Unknown	9	No
AB11	34	F	Disease	10	Yes
AB12	47	M	Optic nerve atrophy	30	Yes
AB13	29	F	Optic nerve tumour	7	Yes
AB14	50	F	Retinal degeneration	30	Yes
AB15	55	F	Disease	19	No
AB16	26	M	Glaucoma	16	Yes
AB17	46	M	Brain tumour	4	Yes
AB18	45	F	Injury	26	No
AB19	23	F	Genetic disease	16	Yes
AB20	31	M	Congenital defect	18	Yes
AB21	26	M	Toxoplasmosis	5	No
AB22	51	M	Retinal degeneration	6	Yes
AB23	20	F	Optic nerve tumour	16	Yes
AB24	37	M	Disease	11	No
AB25	18	F	Optic nerve atrophy	13	Yes
AB26	40	M	Genetic disease	35	Yes
AB27	50	M	Retinal degeneration	42	No
AB28	60	F	Retinal degeneration	59	Yes
AB29	36	M	Optic nerve atrophy	34	Yes

* Declared by the participants

## Appendix 2

Additional analyses on drawing size exclusively considering the drawings produced on foils.

The ANOVA with the within-participant variables of size rank (1 – key vs. 2 – apple vs. 3 – shoe vs. 4 – backpack vs. 5 – dog vs. 6 – floor lamp vs. 7 – car vs. 8 – house) and the between-participant variables of participants’ visual status (congenitally blind vs. adventitiously blind) and order of drawing (on foil first vs. on paper first) showed a significant main effect of size rank, *F*(4.88, 268.58) = 33.33, *p* < .001, *η*_*p*_^*2*^ = .38 – best explained by the linear function, *F*(1, 55) = 119.15, *p* < .001, *η*_*p*_^*2*^ = .68, and a significant interaction between size rank and visual status, *F*(4.88, 268.58) = 2.64, *p* = .025, *η*_*p*_^*2*^ = .05. The remaining main effects and interactions did not reach significance (all ps > .317). To dissect the interaction obtained, we calculated follow-up ANOVAs for each visual status (congenitally blind and adventitiously blind) separately with the within-participant variables of size rank. Among congenitally blind participants, the main effect of size rank was significant, *F*(4.59, 128.42) = 21.66, *p* < .001, *η*_*p*_^*2*^ = .44, and best explained by the linear function, *F*(1, 28) = 83.96, *p* < .001, *η*_*p*_^*2*^ = .75. Similarly, in the adventitiously blind group, the main effect of size rank was also significant, *F*(4.19, 113.02) = 14.83, *p* < .001, *η*_*p*_^*2*^ = .35, and best explained by a linear function *F*(1, 27) = 43.63, *p* < .001, η_*p*_^*2*^ = .62. In both groups of participants, the size of the drawing increased as the size rank value increased (see Table 2).

## Footnotes

1 Although there are opinions in the literature that the imagery of blind people (including those with congenital blindness) is vision-like (e.g., Renzi et al., 2013) or that their memory representations are visuo-spatial (Cattaneo et al., 2008), the metaphor of the mind’s hand (Blanco & Travieso, 2003; Szubielska, 2021) seems more accurate in this case than the mind’s eye metaphor.
